# Pension funds and fossil fuel phase-out: historical developments and limitations of pension climate strategies

**DOI:** 10.1007/s10784-024-09626-0

**Published:** 2024-03-18

**Authors:** Clara McDonnell

**Affiliations:** Governance and Inclusive Development Research Group, Department of Geography, Planning and International Development, University of Amsterdam, Nieuwe Achtergracht 166, 1018WV Amsterdam, The Netherlands

**Keywords:** Pension funds, Fossil fuels, Climate, Supply-side, Divestment, Engagement, Shareholder

## Abstract

Despite the decades of international climate negotiations and several landmark agreements, global efforts to date to restrict fossil fuel production in line with climate targets have been unsuccessful. As national and international policies continue to fall short of phasing out fossil fuels, increasing attention has been paid to non-state actors, like pension funds, as a potential source of more ambitious climate action. As major asset owners, large shareholders in fossil fuel companies, and historically activist investors, pension funds are theoretically well-placed to contribute to phasing out fossil fuels. Despite growing recognition of this potential role for pension funds and other major investors in climate change mitigation, there has been little attention to pension funds’ historical record on climate change, or to how their climate strategies have developed and changed over time. This paper examines how the climate strategies of the largest US and European pension funds have evolved in relation to key developments in international climate agreements and the extent to which these strategies contribute to restricting fossil fuel supply. Through an analysis of the annual, governance, and sustainability reports of 6 pension funds from 1997 to 2022, we examine the strategies pension funds have adopted to address both climate change and fossil fuels. Pension funds have demonstrated responsiveness to the signals of international climate agreements, adopting a range of strategies with respect to climate change (amongst others, integrating ESG principles, increasing their sustainable investments, and setting net zero goals). Their explicit attention to fossil fuels and contribution to supply-side interventions take the form of systematic shareholder engagement, (selective) divestment, and lobbying policymakers. While pension fund climate action is growing , the ambition of their strategies is not aligned with a rapid fossil fuel phaseout; their efforts are often focussed on improving disclosure and transparency and demonstrate complacency with minimal improvements from fossil fuel companies. If pension funds are to significantly contribute to phasing out fossil fuels, redefining pension fund responsibilities and the traditional shareholder role will likely be required.

## Introduction 

Thirty years after the adoption of the UN Framework Convention on Climate Change (UNFCCC) at the 1992 Earth Summit in Rio de Janeiro, it is clear that the success of the global framework to sufficiently address climate change has been limited. Although predictions for planetary warming are not as dire as once predicted and several landmark agreements have been reached, the world nonetheless faces an increasingly urgent climate crisis (Wallace-Wells, 2022). Phasing out fossil fuels remains a critical priority for mitigating the worst impacts of climate change, as approximately 40% of developed fossil fuel reserves (and much more of overall known reserves) must remain unextracted to limit global warming to 1.5 °C (Trout et al., 2022; Welsby et al., 2021). The outcomes of COP28, while demonstrating progress at the international level, nonetheless fall far short of the level and ambition of action needed for a rapid fossil fuel phase-out (Carbon Brief, 2023). Given the insufficient progress of international climate negotiations, pension funds and other institutional investors are increasingly seen as a potential ‘second-best’ source of climate action (Gunningham, 2020). Due to both their size (pension assets amount to about $57 trillion globally) and their ongoing investment in fossil fuel companies, pensions could contribute to limiting fossil fuel extraction and production as powerful shareholders or potential sources of finance for green projects (Thinking Ahead Institute, 2022a). Recent studies have begun to examine the role of pension funds in climate mitigation, mostly focussing on the carbon or fossil-intensity of their investments, or on the extent to which they are decarbonising their portfolios (e.g. Boermans & Galema, 2019; Egli et al., 2022), while others examine their recent approaches to climate action (e.g. Krueger et al., 2020; Rempel & Gupta, 2020). Although the potential for investors to contribute to climate change mitigation and fossil fuel phase-out has been noted (Dordi et al., 2022; Gunningham, 2020; Henderson, 2020), there has been little work that examines how pension fund climate strategies have evolved throughout the decades of climate negotiations or on their contributions to supply-side fossil fuel restrictions. Greater understanding of the range of strategies pension funds use, as well as exploration of their consistency, ambition, and effectiveness over time, could contribute to better conceptualising the possibilities and limitations of pension funds as agents of climate change mitigation. Thus, this paper asks: how have the climate strategies of the largest US and European pension funds evolved in relation to key developments in international climate agreements and how could these strategies contribute to limiting fossil fuel supply? The article first reviews the role of finance and investors in international climate agreements (2.1) and supply-side fossil fuel restriction (2.2), presents the methods (3) and results (4) before discussing and presenting conclusions (5–6).

## Defining pension fund roles in climate mitigation

There are two broad lines under which we can consider the role of pension funds in contributing to climate action. First, pension funds, as large institutional investors, are a potential source of the much-needed capital for climate mitigation and adaptation. This type of financing typically falls under the label of ‘climate finance’ and is the main way that the financial sector is directly addressed by international agreements. Second, pension funds, as investors in fossil fuels and other polluting sectors, may also have a role to play in limiting the climate damage enabled by such companies, through their role as investors. Although this role is not spelled out by climate agreements, it is implicitly included in the Paris Agreement, which commits in Article 2c to “making finance flows consistent with a pathway towards low greenhouse gas emissions and climate-resilient development” (UNFCCC, 2015). Through their role as major shareholders, pension funds may be able to contribute to supply-side restrictions on fossil fuels, a necessary step for mitigating climate change which is nonetheless missing from international agreements (van Asselt, 2021; van Asselt & Kulovesi, 2017). These two lines of action are discussed in more depth below.

### The role of finance in international climate agreements

Prior to 2009, climate negotiations focussed more on the role of ‘funding’ than finance, which consisted of short-term, public, project-based finance, while a range of market mechanisms were elaborated in the 1997 Kyoto Protocol as policy options for countries to adopt (Gupta, 2010; Peake & Ekins, 2017). During this phase, investors played a relatively peripheral role; they were potential participants in market mechanisms, such as emissions trading schemes, but were not considered as a direct source of climate finance. The 2009 Copenhagen Accord marked a turning point in the approach taken by the international governance regime. Top-down approaches to determining emissions reduction targets were replaced by decentralised and nationally-determined targets, while a common global target was adopted (limiting warming to 2°C) (Bäckstrand et al., 2017; Kuyper et al., 2018). There was also a growing number of voluntary climate initiatives coming from non-state actors (NGOs, investors, companies, amongst others), many of which developed in response to inadequate action at the national and UNFCCC levels (Bäckstrand et al., 2017). There was a shift towards the provision of long-term finance, and the term ‘climate finance’ came into wider use, defined by the UNFCCC as “local, national, or transnational financing—drawn from public, private and alternative sources of financing—that seeks to support mitigation and adaptation actions that will address climate change” (Bäckstrand et al., 2017; Peake & Ekins, 2017; UNFCCC, n.d., para. 1). The Copenhagen Accord quantified an expected target ($100 billion per year by 2020) for climate finance provided by developed countries to address the needs of developing countries and established the Green Climate Fund to facilitate funding. Bracking (2019, p. 712) argues that this shift was also marked by an increasing financialisation of climate finance, drawing “capital markets proper into environment, conservation and climate management” through products such as green bonds and indices, as well as through more complicated asset forms, such as catastrophe or weather bonds.

The 2015 Paris Agreement institutionalised the commitment of climate finance from developed countries to developing countries in a treaty, recognising that the level of finance should “represent a progression beyond previous efforts” (UNFCCC, 2015, p. 13). The growing neoliberalisation and financialisation of the types of instruments and arrangements used to secure climate finance indicates an increasing orientation towards private finance and debt-based finance and a potential increasing importance for actors like institutional investors in delivering climate finance commitments (Bracking, 2019; Bracking & Leffel, 2021). The Paris Agreement also sent a stronger signal to global markets and institutional investors than previous agreements, due to its inclusion of a long-term emissions goal, though this was weakened by the lack of specifics with respect to the time frame or pathways (Falkner, 2016). With the development of international climate agreements, the role for institutional investors as a source of climate mitigation and adaptation finance has become increasingly institutionalised, while investors themselves have become more active amongst the proliferation of non-state climate initiatives occurring alongside the formal climate negotiations (Bäckstrand et al., 2017; Banda, 2018).

### Shareholder contributions to supply-side interventions

While international climate negotiations and agreements have integrated investors as a potential source of finance for mitigation and adaptation projects, they largely do not address the role of investors as funders of climate-damaging sectors, including fossil fuels. The Paris Agreement’s commitment to aligning financial flows with climate goals implies that investors will need to make significant changes, however, there are no details or concrete steps specified in the agreement. The literature on fossil fuel supply-side interventions, as well as the literature on shareholder activism and climate change, provide important contributions to develop a more complete picture of the role investors play in climate action. Calls for supply-side fossil fuel policy argue that the decades of focus on demand-side policy have had limited success and that merely scaling up alternative energy sources (through e.g. greater private investment) will be insufficient for phasing out fossil fuels at the rate necessary for meeting climate goals (Lazarus & van Asselt, 2018; Piggot et al., 2018; York & Bell, 2019).

Pension funds, as well as other large investors, have an indirect, but potentially important role to play in limiting fossil fuel supply. As major shareholders, pension funds may be able to influence the corporate strategy adopted by the fossil fuel companies they invest in, although this influence is generally limited to publicly listed companies (Christophers, 2021). Divestment, selling off fossil fuel assets, is often cited as a supply-side measure adopted by investors (Lazarus & van Asselt, 2018; Pellegrini & Arsel, 2022; Piggot et al., 2020). However, while the divestment movement is motivated by the goal to leave fossil fuel reserves untouched, the contribution of divestment to actual limitations on fossil fuel supply is less clear. While selling off fossil fuel assets may only indirectly impact the divested companies (through e.g. their social reputation), other strategies adopted by the divestment movement have had more concrete impacts on limiting fossil fuel extraction. For example, Curran (2020) documents how activist efforts to lobby financial institutions to limit financing or insurance for a specific project (Adani’s Carmichael mine in Australia) were successful in limiting the scale of the project (although not in cancelling it completely).

As an alternative strategy, or in addition to divestment, investors may also engage as shareholders with company management to attempt to influence companies to change from within. Shareholder engagement encompasses a range of public and private strategies, including private discussions with company management, public statements, or proxy voting. The content of shareholder engagements can often be opaque. Although certain forms of engagement, such as proposing and voting on shareholder resolutions, are publicly available, some of the strategies considered more effective–especially closed-door meetings with management–are private (Azar et al., 2021; Baines & Hager, 2022). Although investors may disclose the topics of their private engagement with companies, the specifics of those discussions are generally unknown. Investor coalitions like Climate Action 100 + (CA100 +) or the Principles for Responsible Investment (PRI) have initiated more formal collective investor engagement with the fossil fuel sector as well as other major emitters and provide some insights into the requests investors make of companies. While these efforts have achieved some incremental steps from fossil fuel companies (such as adopting long term net-zero goals), they have not yet resulted in concrete commitments to limit fossil fuel extraction and production (McDonnell et al., 2022). The literature on shareholder engagement as a form of climate action has generally focussed on the relationship between investors and high-emitting companies; however, there are arguments that investor engagement with other actors (e.g. asset managers, fossil fuel financiers) could contribute to mitigating fossil fuel production (McDonnell & Gupta, 2023; Urban & Wójcik, 2019).

## Methods

To focus on pensions with the most potential leverage over the fossil fuel sector, a sample of 3 of the largest public funds from both the US and the EU was selected. The US and the EU are amongst the largest global pension markets (holding about 62 and 6% of pension assets respectively) and are home to some of the largest global pension funds (Thinking Ahead Institute, 2022a). US and European pension funds have a history of being active owners and adopting social or environmental criteria into their investment strategies (Clark & Hebb, 2005). The EU and European countries are also global leaders in adopting green or sustainable financial regulation (Egli et al., 2022; Steffen, 2021). The selected sample of pension funds may thus represent the vanguard of investor climate action. By focussing on those pensions most likely to have adopted ambitious climate strategies, I hope to assess both the historical development of those strategies and the gaps that remain for investors to meaningfully align with a principle of leaving fossil fuels underground. Table 1 presents the 6 pension funds studied in this paper.

**Table 1:** Pension sample

Fund	Year founded	Country	Total Assets (in USD million)^a^	Global Rank (size)	Fossil fuel investments (in USD million)^b^
California Public Employees Retirement System (CalPERS)	1932	U.S	496,820	6	13,007
California State Teachers Retirement System (CalSTRS)	1913	U.S	313,940	11	7,033
New York State Common Retirement Fund (NYSCRF)	1921	U.S	267,756	12	5,591
Algemeen Burgerlijk Pensioenfonds (ABP)	1922	Netherlands	630,358	5	6,047
Pensioenfonds Zorg & Welzijn (PFZW)	1969	Netherlands	315,467	10	3,875
Arbejdmarkedets Tillægspension (ATP)	1964	Denmark	155,351	24	205

^a^As of December 2021 (Thinking Ahead Institute, 2022b)

^b^Total value of shares and bonds of oil, gas, and coal companies, as of January 2023 (Urgewald, 2023)

To analyse pension climate strategies, pension fund publications (e.g. annual reports, sustainability reports) are used. Relevant documents were accessed from pension websites. Most of the sampled pensions keep documents from several prior years accessible on their websites. Several strategies were used to access earlier documents. Earlier versions of pension websites were accessed through the Internet Archive,^1^ a non-profit digital archive of the Internet. Some annual reports and other documents were accessible through databases such as Public Plans Data.^2^ Finally, requests were made to all pension funds in the sample for annual reports and sustainability reports for the period 1992–2022, and in several cases, pension funds provided reports. Although all the pensions produce annual reports, the ways they report on sustainability initiatives vary. Some produce sustainability or climate reports, while others incorporate sustainability concerns into their corporate governance reporting or annual reports. In several cases, snapshots of pension web pages were collected, when containing relevant information not included in available publications.

326 documents were included for analysis; the number and type of documents collected for each pension are listed in the Appendix. Documents in English and Dutch were included, since many documents for ABP and PFZW were available only in Dutch. To facilitate analysis of a large number of documents, all publications were imported into Atlas.ti and the ‘search and code’ function was used to identify relevant passages in the reports for analysis. An extensive list of search terms was generated to identify text segments related to climate, sustainability, or fossil fuels (see Appendix). Segments were then coded inductively, using an open coding process guided by the research question to identify types of climate action pension funds adopted, or pension stances on climate issues identified. After this first round of coding was completed, the documents and relevant sections of the text were then reviewed again to add context and nuance as well as to develop a timeline of key actions and developments for each pension fund. The timelines were then compared to identify common themes and strategies across pension funds. The results are presented as a chronological narrative. While the results often summarise trends identified across the pension funds, further detail on key developments and steps taken by each pension fund are illustrated by detailed timelines for each period identified. While the potential biases of relying on pension self-reported information must be accounted for, annual reports are also considered a reliable and consistent source for information on investor activity and have been used in other sectors to critically assess corporate climate strategies (Li et al., 2022; Megura & Gunderson, 2022). This focus gives extensive insight into the types of strategies adopted by pension funds; however, it means that full understanding of the context and factors shaping such strategies may be limited. Further research into the political, economic, and policy context surrounding pension fund climate action could enrich the findings of this paper.

## Results 

### Kyoto to Copenhagen: ramping up investor action (1997–2009) 

#### Emergence of sustainability strategies

Prior to the 1997 Kyoto Agreement, and the years following, there is little evidence of explicit investor attention to climate change, although pension funds were adopting responsible investment and stewardship programmemes since the mid-1980s. Explicit attention to climate change starts to become visible in the early 2000s. The increasing attention to climate frequently takes the form of joining new investor initiatives focussed on climate and responsible investment. All 6 pension funds endorsed the CDP’s (formerly, Carbon Disclosure Project) annual questionnaires sent to major carbon emitters, asking for disclosure of their emissions. All the funds also joined onto the PRI, in some cases as founding members, and the US pensions joined Ceres and its investor branch, the Investor Network on Climate Risk (INCR).

From the early 2000s, many of the pensions started to implement environment- or climate-focussed programmes, including strategies for engaging high-polluting companies, as well as for increasing environmentally-oriented investments. CalPERS and CalSTRs adopted environmental strategies upon request from the California State Treasurer in 2004, which involved developing a corporate governance strategy to encourage company disclosure of environmental risks (including climate risks), increasing investments in clean technologies and environmentally friendly companies by $500 million from each pension fund, and auditing real estate assets for energy efficiency and green standards (CalPERS, 2005a, 2012). Both pensions were amongst the founders of the Climate Risk Disclosure Initiative in 2005, which developed a framework for the climate-related disclosures expected from companies (Climate Risk Disclosure Initiative, 2006). In addition, CalPERS submitted several shareholder resolutions at auto companies and pledged to develop engagement strategies to target the auto companies and utilities, given their expectations that climate policy would impact these sectors (CalPERS, 2005a). A report sponsored by CalSTRS in coordination with the CDP states that electric utilities are “the most carbon intensive sector and has therefore been one of the first to feel the effects of environmental regulations and the pricing of carbon emissions” (Trucost, 2006, p. 1). Efforts to engage the auto industry cite the need for compliance with California GHG standards (CalPERS, 2005a). Programmes adopted by ABP and PFZW around the same period focussed on integrating ESG criteria into their investment practises (at first in equity portfolios, with the ambition to expand to other sectors, increasing sustainable investments, and integrating ESG into their assessments of external asset managers) (ABP, 2007; PGGM, 2006).

#### Investor calls for climate policy

In the years leading up to the 2009 UN Conference of Parties (COP) meeting in Copenhagen, most of the pensions publicly call for a climate agreement or issue statements of support for climate policy. ABP discusses in their 2008 annual report their support, along with other major global investors, for the Investor Statement on a Global Agreement on Climate, released during COP14 in Poznań. ABP highlights the need for a consistent policy signal on climate:

“Sudden policy changes can erode the value of existing investments in areas such as renewable energy. It is also important to keep future investment opportunities attractive in the long term. It is important that existing schemes do not change too often or too much in form and scope” (ABP, 2008, p. 76).

PFZW discusses meeting with EU MEPs to call for a strong agreement in Copenhagen (PFZW, 2009). The US pension funds all discuss lobbying the US Securities and Exchange Commission (SEC) for stricter environmental and climate disclosure requirements for companies (CalPERS, 2004; NYSLRS, 2009).

#### Initial fossil fuel engagements

**Fig. 1:**
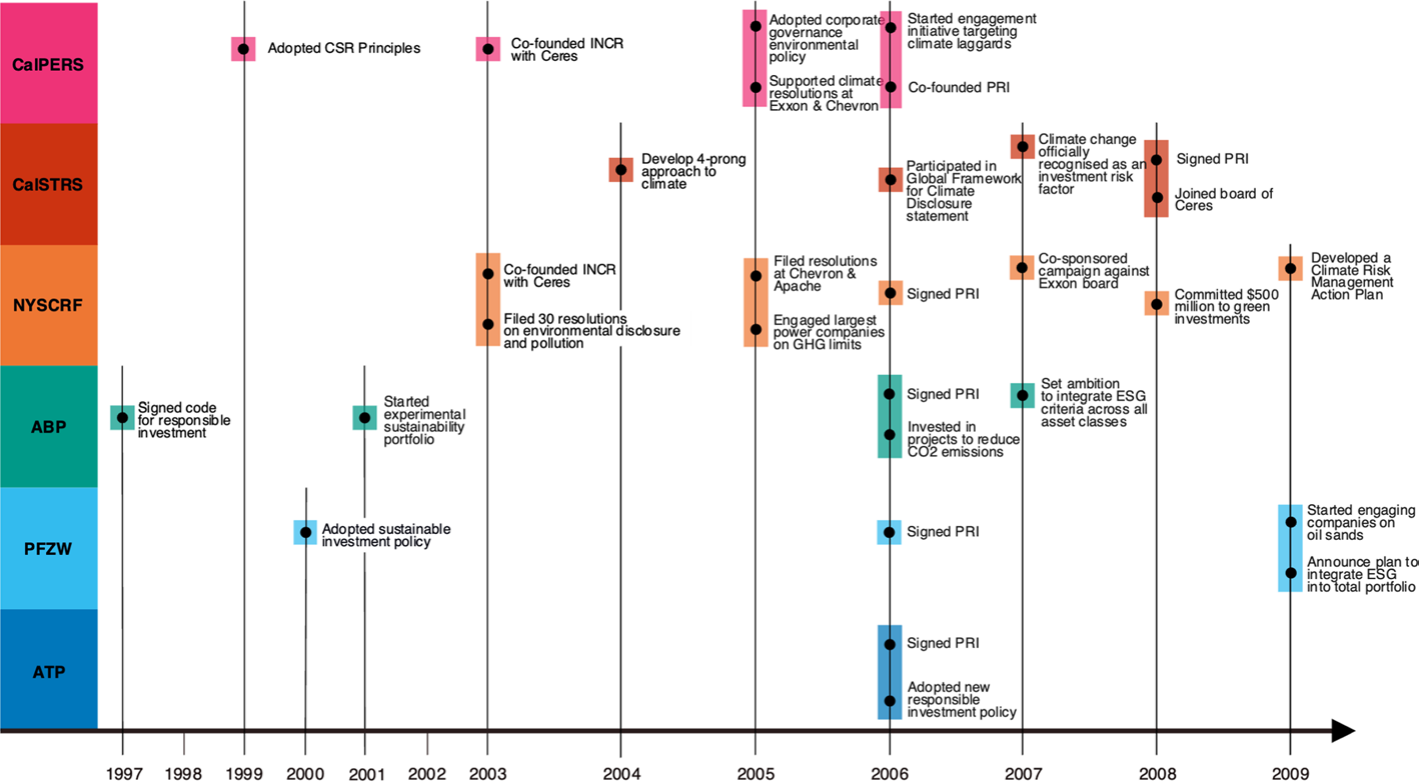
Key events and developments in pension fund climate strategies (1997–2009)

During this initial phase of pension fund attention to climate change, there is some evidence of shareholder engagement with the fossil fuel industry, although it is limited to a few select companies and often focussed on environmental damages or human rights abuses. In 2005, NYSCRF co-sponsored a resolution at ChevronTexaco, supported by CalPERS, asking for information on how the company planned to address environmental and health concerns associated with oil spills and contamination around their sites in Ecuador (CalPERS, 2005b; NYSLRS, 2005). ATP decided in 2007 to exclude oil and gas companies extracting in Myanmar (they ended this exclusion policy in 2014) (ATP, 2015a). There was some limited engagement on the climate impacts of fossil fuel operations, which largely centred on disclosure. NYSCRF filed a resolution at Apache Corporation (a US oil and gas company), requesting that the company “report on and develop ways to mitigate risks of carbon emissions,” withdrawing it after the company agreed to produce such reporting (NYSLRS, 2005, p. 60). In 2007, they submitted resolutions at two coal companies, asking for reports on their response to rising pressures to reduce emissions. CalPERS supported another 2005 resolution at ExxonMobil asking for reporting on compliance with the Kyoto Protocol and included the oil and gas sector in their strategy to target laggards in environmental disclosure (CalPERS, 2005b, 2006). Other fossil fuel companies (e.g. Shell, BP) are highlighted as good examples of compliance with disclosure requests (CalPERS, 2006). In one particularly prescient case, CalSTRS and NYSCRF did not support reappointment of a board director at Exxon due to the company’s inaction on climate risks, a strategy that doesn’t appear again until after the Paris Agreement (see 4.3.2) (NYSLRS, 2007). PFZW started engagement with Canadian oil sands companies in 2009, although little detail is given on the content of those engagements (PFZW, 2009). During this period, pensions express some concern over the investment risks associated with fossil fuel investments, largely due to the potential for regulation. For example, as part of their motivation for engaging companies on environmental disclosure, CalPERS cites research which suggests that “shareholders in leading oil and gas companies could lose as much as 5 to 7 percent of the value of their investments because of regulatory and other efforts to respond to climate change” (CalPERS, 2004, p. 8). However, they also emphasise the expectation that demand for fossil fuels will continue for the foreseeable future. While discussing their investments in sustainable energy sources, ABP states that while they don’t expect these forms to replace the need for fossil fuels, they may be able to reduce demand (ABP, 2007). Fig 1 illustrates, by pension fund, the key actions taken by each fund during the period discussed above.

### Copenhagen to Paris (2010–2015) 

#### Maturing climate strategies

In the years leading up to the Paris Agreement, there is a notable uptick in investor attention to climate and a refinement and expansion of many of the strategies that were introduced in previous years. In 2011, the board of CalPERS approved a ‘total fund’ approach to ESG, integrating ESG criteria into the investment and engagement strategies for all asset classes. Climate change was one of three key focus areas for the strategy, which they planned to address by engaging with a ‘focus list’ of companies, developing a consistent approach to ask internal and external investment managers to consider environmental issues, and scaling up investments in clean energy and technology (CalPERS, 2012). In 2012, PGGM (the asset manager for PFZW) developed an ESG index for passively managed equity, which excludes the bottom 10% of companies in each sector that perform the worst on ESG criteria (PGGM, 2013). Multiple pensions collaborated with Mercer to publish reports in 2010 and 2015 which developed methods for assessing climate risks of different asset classes and assessing overall portfolio resilience based on varying climate scenarios (CalPERS, 2012; CalSTRS, 2016a; Mercer, 2015). In 2014–2015, most of the pension funds made commitments to scale up their climate-related investments. CalSTRS explicitly ties their green investment to the Paris Agreement, saying “in December 2015, we were one of the first large institutional investors to commit to the COP21 Paris Pledge for Action” with a pledge to “increase clean energy and technology investments from $1.4 billion to $3.7 billion by the year 2019” (CalSTRS, 2016a, p. 5).

The pensions also continued to lobby for climate policy in coordination with the COP process, issuing statements in multiple years before COP sessions, calling for global leaders to adopt climate policies (ABP, 2010; ATP, 2014). In addition to general calls for a climate agreement and commitments to emissions reductions, there were calls for implementing carbon pricing, supporting investment and innovation for sustainable technologies and energy, and critical scrutiny of supervision of financial institutions that hinder investments in green growth (PGGM, 2014). Pensions also began to attend and participate in international climate meetings. CalPERS participated in the 2011 COP16 in Durban, where they attended several high-level meetings and contributed to producing an Investor Action Plan on Climate Change Risks and Opportunities which “calls for effective policies on climate change and clean energy, improved regulation and transparent reporting” (CalPERS, 2012, p. 27). CalPERS, NYSCRF, ABP and PFZW all discuss attending COP21 in Paris, while CalSTRS and ATP indicated their support for the adoption of a climate agreement.

Finally, ABP and PFZW were at the forefront of measuring the carbon footprint of their portfolios and making commitments to reduce it. PGGM signed the Montreal Carbon Pledge in 2014, in which investors commit to monitor and disclose the carbon emissions of their investment portfolios (PGGM, 2014), and set a target of halving the emissions of their public equity portfolio by 2020 (PFZW, 2014). In order to meet the target, they adopted a policy of selective divestment, selling shares of the top polluting companies in the most polluting industries and reinvesting that capital in less carbon-intensive companies in the same sector (PGGM, 2015). ABP started calculating their portfolio emissions in 2013 and in 2015 made a commitment to reduce the carbon emissions of their equity portfolio by 25% by 2020 (ABP, 2014, 2015b). In 2015, CalPERs also signed the Montreal Carbon Pledge, although without any concrete commitments to reduce emissions (CalPERS, 2015).

#### Formalising fossil fuel engagement strategies

In the period between the Copenhagen Accord and the Paris Agreement, pensions rapidly expanded their focus on and engagement with companies in the fossil fuel sector. As in the previous period, much of this engagement was in response to concerns around safety or environmental impacts after disasters or in response to new unconventional forms of extraction. CalPERS highlights select engagements, including their engagement with BP on their environmental risk management practises after the 2010 Deepwater Horizon spill and with Massey Energy after a 2010 disaster which killed 29 workers (CalPERS, 2012). ABP reduced their investments in BP after the disaster, while NYSCRF joined a group of investors in writing to 27 major oil and gas companies asking them to review the safety of their offshore drilling operations (ABP, 2010; NYSLRS, 2011). They also joined a securities fraud lawsuit against BP which argued that the company misled investors on the safety risks of offshore drilling (NYSLRS, 2011). In 2010, NYSCRF filed shareholder proposals at oil and gas companies asking for disclosure on the risks (environmental and regulatory) associated with fracking (NYSLRS, 2010). The same year ABP engaged with oil and gas companies on the risks associated with oil sands extraction, as well as with Shell on pollution of the Niger Delta (ABP, 2010). In 2011, NYSCRF submitted several resolutions asking coal companies to report on the climate impacts of their operations and asking oil and mining companies to appoint directors to their board with environmental expertise (NYSLRS, 2011). CalSTRS discusses working with the INCR to lobby securities regulators in the US and Canada to issue formal guidance for oil and gas companies on climate disclosure, as well disclosure and oversight on the risks of the extractive processes discussed above (CalSTRS, 2012). PFZW engaged fossil fuel companies on risk management, but also on the need to increase their investment in clean energy (PGGM, 2012).

**Fig. 2:**
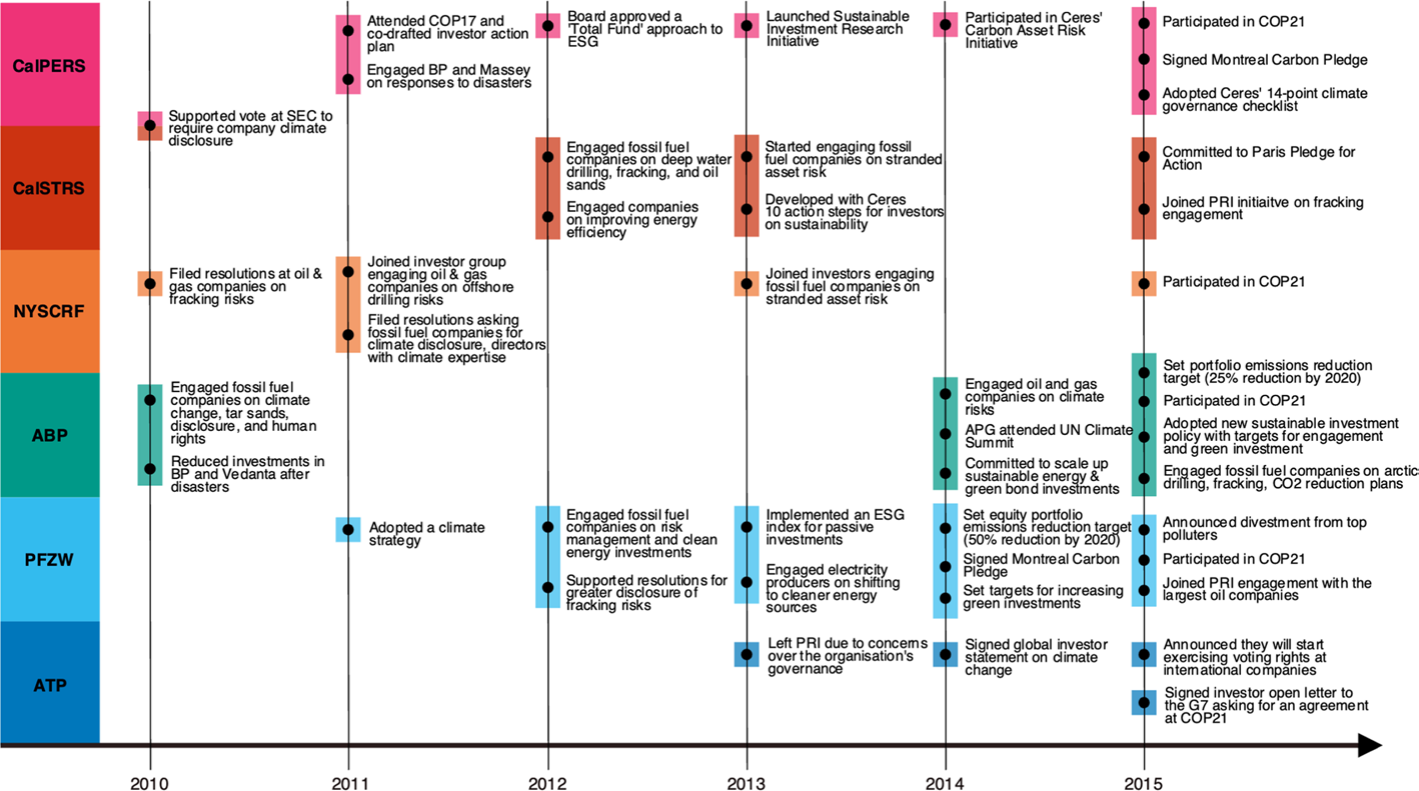
Key events and developments in pension fund climate strategies (2010–2015)

In 2013, while continuing engagements on the environmental risks of extraction, pensions also began to engage fossil fuel companies on their reserve valuation. This was largely due to the influence of the Carbon Tracker Initiative’s 2011 *Unburnable Carbon* report, which argued that many fossil fuel reserves were overvalued, given the need to leave large portions of the reserves unextracted in order to meet climate goals. CalPERS, CalSTRS, and NYSCRF joined an initiative led by Ceres, the Carbon Asset Risk Initiative, which called on 45 oil and gas companies to disclose risk management strategies for a 2 °C climate scenario, as well as emissions associated with operations and reserves, how and why companies are dedicating capital expenditure towards the exploration and development of new reserves, and risks of stringent climate policy (CalPERS, 2014; CalSTRS, 2013, 2014b; Office of the New York State Comptroller, 2013). In 2015, ABP started to implement minimum conditions for the companies they invest in and centred their engagements with high polluters around those criteria. They include: disclosing emissions, reducing emissions by 25% by 2020, dedicating resources towards developing climate solutions, and for energy companies, aligning their business with climate policy, shifting as much as possible away from oil and coal and towards gas, and adopting safety protocols (ABP, 2015a). Fig. 2 illustrates key actions taken by each pension fund during the period between the Copenhagen Accord and the Paris Agreement.

### Paris to present (2016–2022) 

#### Growing standardisation of disclosure and target-setting

Post 2015, there is a fresh wave of investor activity based around achieving the goals of the Paris Agreement. The Financial Stability Board created in 2015 the Task Force on Climate-Related Financial Disclosures (TCFD), with the intent of identifying the financial risks of climate change and encouraging consistent disclosure of risks. In 2017, they published their recommendations for disclosure, which most of the pensions explicitly endorse and incorporate into their engagements on climate-related disclosure. Several also adopted the disclosure recommendations for asset owners and disclose their carbon emissions and climate risk analysis in line with the TCFD framework (ATP, 2017b; CalSTRS, 2019; NYSLRS, 2018; PFZW, 2017). All the pensions joined CA100 + , which unites investors to engage the 171 top emitting companies globally on climate issues, and they conduct much of their engagement with fossil fuel companies through that platform. Following the IPCC’s (2018) special report on 1.5 °C, most pensions began to incorporate net zero by 2050 as the long-term emissions reduction target expected of companies they engage with, as well as ultimately of their own portfolios. CalPERS joined the Net Zero Asset Owner Alliance (NZAOA) in 2019, while CalSTRS committed to a net zero action plan in 2021 as part of the UN Race to Zero ahead of COP26 in Glasgow (CalPERS, 2019; CalSTRS, 2021). NYSCRF adopted a net zero by 2040 goal for their portfolio emissions in 2020 (NYSLRS, 2021). The three EU funds have recently adopted net zero by 2050 goals, and have set short term targets, ABP and PFZW aiming to reduce emissions by 50% by 2030 and ATP aiming for 70% by 2030 (ABP, 2022a; ATP, 2022; PFZW, 2022). ATP has been the most vocally critical about the limitations of setting emissions reduction targets for climate mitigation and about the value of delimiting green versus dirty investments. They argue that while there is value in tracking emissions, changes in emissions levels of an investor portfolio does not translate to real emissions reductions (ATP, 2017a). They also discuss the limitations of using emissions alone to manage climate risks. They give examples of wind turbine manufacturers, who may have high emissions, but also play a critical role in the energy transition and often discuss the Danish company Ørsted (formerly DONG Energy) as a ‘dirty’ investment which succeeded in transforming into a green company. By excluding such companies in an aim to reduce portfolio emissions, they argue that investors “deny themselves the opportunity of contributing to the green transformation of energy production” (ATP, 2017a, p. 22).

#### Fossil fuel engagement wins

Post-Paris, pensions continued to engage fossil fuel companies and achieved several notable shareholder wins at oil and gas majors. Shareholder resolutions still focussed on disclosure, although they gained more success than in prior years. In 2016, CalPERS reported wins at BP and Shell as the companies agreed to back shareholder proposals on climate risk disclosure and cite investments both companies had made in alternative energies (CalPERS & CalSTRS, 2016). CalSTRS, motivated by proposed EPA regulation on methane emissions capture, began an engagement programme focussed on asking oil and gas companies to disclose their methane emissions and strategies to manage them (CalSTRS, 2016b). CalSTRS describes 2017 as a “watershed year for climate change proposals” as proposals at both Exxon and Occidental Petroleum received majority shareholder approval, largely due to the support of the major American asset managers (CalSTRS, 2017b, p. 6; PGGM, 2017). The proposals asked the companies to disclose the impact that a 2 °C scenario would have on their business. In 2016, Dutch activist investor group Follow This began submitting shareholder resolutions at Shell (and later expanding to other oil and gas majors), asking the company to cease exploration for new reserves and to set concrete emissions reduction targets. Both Dutch pensions did not support resolutions from Follow This in 2016 or subsequent years, considering them to be too demanding on the company. ABP stated in 2016: “We think the Board of Directors and not the shareholders should decide on Shell’s transitional strategy. We do however, welcome the way in which the initiative takers are contributing to awareness of climate change” (ABP, 2016, p. 28). In following years, they abstained from voting on the resolutions, arguing that given Shell’s willingness to set CO2 reduction targets (2018) and adopt a net zero target (2020), the resolutions were not necessary (ABP, 2018, 2020). ATP did support the resolution in 2018, since Shell had announced ambitions, but had not yet set concrete targets (ATP, 2018).

From 2017 to 2020, investors generally report success in engagements with fossil fuel companies, especially through CA100 + , as many of the majors adopted climate plans and net-zero targets. However, in 2020, investors started to escalate their engagement strategies again, citing a lack of credibility in fossil fuel company climate plans. In 2020, the US pensions all discuss supporting the (successful) effort from activist investment fund Engine No. 1 to reject three of the board’s nominations for directors at Exxon and instead elect candidates with environmental and climate-related expertise. After the Exxon vote, there is a distinct shift in pension engagement strategies towards using votes against directors and board members as a means of escalating unsuccessful engagements. In 2022, CalPERS and CalSTRS state they will vote against directors on climate grounds, CalSTRS committing to vote against directors at the “highest global emitters if they have not published a TCFD-aligned report, disclosed scope 1 and scope 2 emissions, and set appropriate targets to reduce greenhouse gas emissions” (CalSTRS, 2022, p. 11). NYSCRF started voting against directors in 2021, and updated their proxy voting guidelines in 2022 to reflect their expectation of net zero goals and 1.5°C alignment (Office of the New York State Comptroller, 2022). ABP also announced in 2020 their plan to vote against director appointment and remuneration at companies without a CO_2_ reduction target and measures to link executive compensation to climate goals (ABP, 2020). In 2022, PFZW announced that they would support Follow This resolutions, and would be intensively engaging all the oil and gas companies they invest in, with the expectation that they produce Paris-aligned climate plans by the end of 2023 (PFZW, 2022). Companies that do not meet these criteria would be subject to divestment.

#### Progressive divestments

**Fig. 3:**
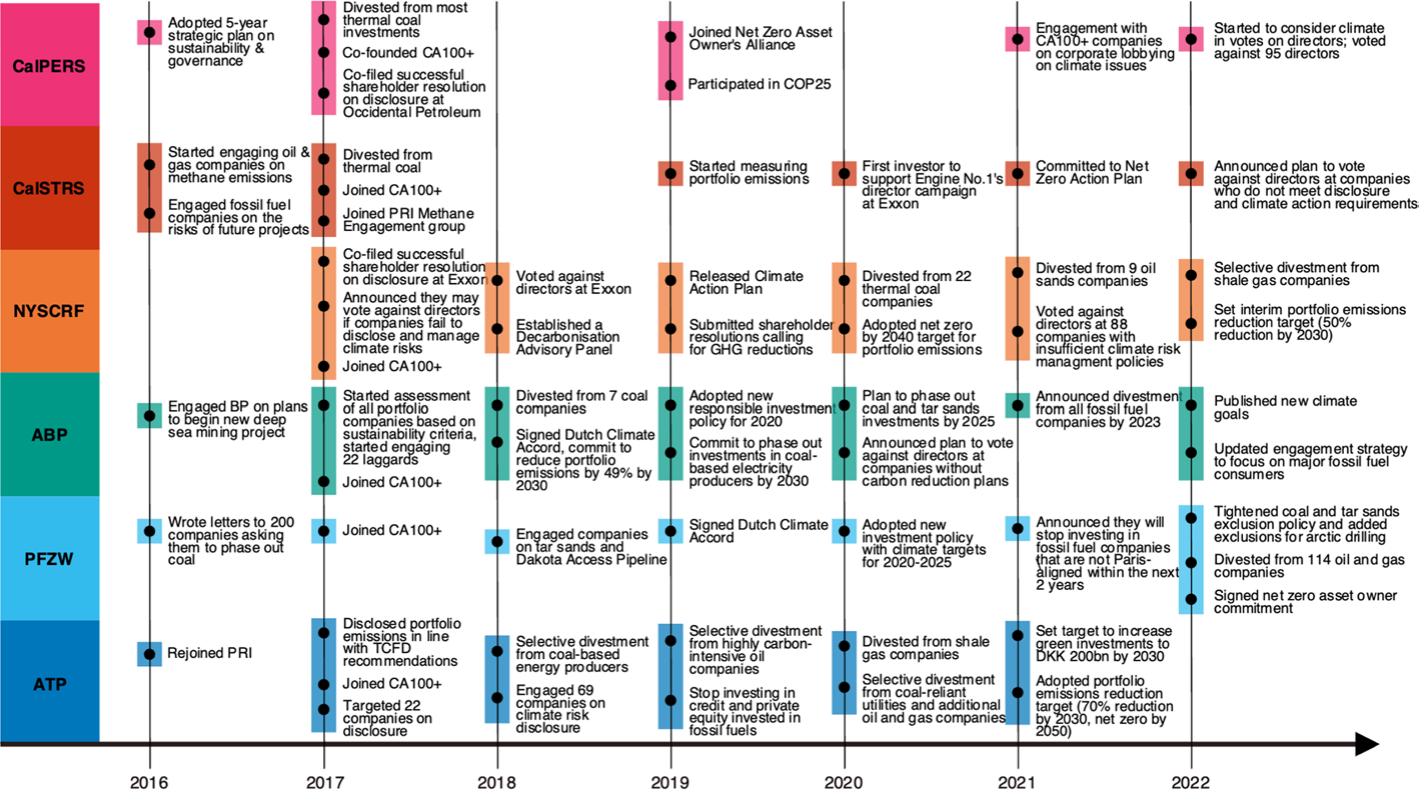
Key events and developments in pension fund climate strategies (2016–2022)

While all the pensions in the sample had previously expressed their preference for shareholder engagement over divestment, post-Paris, many pensions also started to selectively divest from fossil fuel companies. In 2015, California passed the Public Divestiture of Thermal Coal Companies Act (SB 185), which required CalPERS and CalSTRS to “consistent with its fiduciary responsibilities, to identify, engage, and potentially divest from companies” generating 50% or more of their revenue from thermal coal mining (CalPERS, 2017, p. 3). While several companies were considered to have credible transition plans, CalPERS divested from 14 companies, and CalSTRS divested from all thermal coal assets (CalPERS, 2017; CalSTRS, 2015, 2017a). NYSCRF adopted a progressive approach to assessing the risks associated with various types of fossil fuel investments and their transition-readiness. As a result, they decided to divest from companies who could not produce a credible climate plan: 22 thermal coal companies in 2020, 9 oil sands companies in 2021, and select shale gas companies in 2022 (NYSLRS, 2021, 2022). In 2022, they announced plans to begin assessing their investments in integrated oil and gas companies (NYSLRS, 2022). In 2019, ATP decided to stop investments in credit and private equity funds invested in fossil fuel extraction, due to the limited control over these illiquid assets, and “since [they] do not want to be bound for long periods of time to assets that might end up as stranded assets” (ATP, 2019, p. 8). In 2020, they decided to divest from companies with assets they consider to be at a higher risk of becoming stranded, such as shale oil companies (ATP, 2020). PFZW had begun divesting from top polluters in 2015 as part of their emissions reduction strategy. They accelerated their divestments in 2022 by tightening their exclusion policy for thermal coal and oil sands producers and divesting from 114 oil and gas companies which they considered not to be Paris-aligned (PFZW, 2022). ABP is unique amongst the sampled pensions in adopting a full fossil fuel divestment policy. After implementing to partial divestments of companies determined to be ‘laggards’ for several years, in 2021 they committed to sell off all assets in companies that make 1% or more of their revenue from fossil fuel extraction (ABP, 2021). ABP had previously often argued for the value of remaining invested in fossil fuels; the reasoning for their shift in policy is not explained, saying only that they hope to tighten their ambition. Fig. 3 illustrates key actions taken by each pension fund in the years since the Paris Agreement.

## Discussion

This paper aims to examine how pension funds’ climate strategies have developed over time and in relation to international climate agreements, and to expand our understanding of their potential to act as agents of climate mitigation, specifically in restricting fossil fuel supply. This analysis demonstrates a responsiveness from pension funds to international climate negotiations and agreements, as they have often implemented climate strategies in reaction to international agreements. There is some evidence for considering pension funds as initiators of climate action, as many have adopted various climate strategies, such as setting net zero goals, ahead of any legislation requiring their action (though they may be anticipating future policy). Amongst the pension funds studied, there is a relatively strong level of convergence of the strategies used, in contrast to findings from other sectors (e.g. oil and gas majors) which note a marked difference between US and European firms (Herzog-Hawelka & Gupta, 2023; Li et al., 2022). The US pensions demonstrate a relatively earlier involvement in shareholder engagement on climate issues, likely due to the corporate governance movement originating in the US (of which pension funds like CalPERS were key actors), as well as the presence of US-based investor initiatives, such as Ceres (Cheffins, 2015; Lannoo, 1999). European pension funds allocated large portions of their portfolios to international equities later than US funds, likely affecting when they initiated broad shareholder activism programmes (Golka & van der Zwan, 2022; van der Zwan, 2017). However, there is general alignment in pension strategies from the second period identified in this paper, beginning around 2009. Although there are distinctions between the specific steps pensions take, they are frequently members of the same initiatives and engage companies on similar topics. It is only the last period that we see evidence of more ambitious strategies from European funds, especially with respect to their level of fossil fuel divestment and portfolio emissions reduction targets. The introduction of climate-related policy may explain some of this discrepancy. For example, the Dutch funds agreed to more ambitious emissions reduction targets as part of the 2019 Dutch climate accord (Klimaatakkoord), although both funds had adopted portfolio reduction targets before that point. The EU has also introduced multiple sustainability and climate-related legislative initiatives which may encourage greater investor attention to climate change (Kelly, 2021). However, this analysis is limited to several of the largest funds public funds from each region, all of whom demonstrate a significant level of attention to climate change; analysis of the pension sector as a whole may present further regional disparities.

While there is thus evidence for considering pension funds as agents of climate action, examining the effectiveness and ambition of their strategies also reveals important limitations for their potential to contribute to mitigating climate change and phasing out fossil fuels. Although this paper identifies a wide range of climate-related strategies adopted by pensions (amongst others, integrating ESG principles into their investment and governance policies, expanding sustainable investments, joining industry initiatives), here I critically examine in more depth those with the most direct relevance for fossil fuel supply-side restrictions: shareholder engagement with portfolio companies, divestment, and lobbying policymakers.

Pension funds’ shareholder engagement strategies have, for the last decade, targeted fossil fuel supply-side actors. Early shareholder engagement on climate focussed largely on the demand side, targeting the major consumers of fossil fuels, namely utility, power, and auto companies. Although pensions engaged fossil fuel companies selectively since the early 2000s (largely on human rights concerns and in response to cases of environmental damage), a notable shift occurs in 2013, when, with the popularisation of ‘Stranded Asset Theory’, pensions begin to explicitly engage fossil fuel companies on questions of fossil fuel supply and the potential stranding of their reserves (CalSTRS, 2013). This shift aligns with greater public attention to the idea of the need to restrict fossil fuel use to meet climate goals, popularised by actors including the Carbon Tracker Initiative and the divestment movement (Ayling & Gunningham, 2017). While pension funds begin to question fossil fuel companies on the credibility of their business plans within the context of climate change, they also appear to accept the narratives presented by fossil fuel companies at the time. For example, CalSTRS presents as a satisfactory engagement result: “The early assessment of this engagement is that fossil fuel producers adhere to strict Securities and Exchange rules on reserve valuation and that environmental-related risks, such as climate change, are continuously evaluated and ways to mitigate carbon emissions are being actively pursued” (CalSTRS, 2013, p. 19). The pattern of increasing pension fund scrutiny of fossil fuel companies, while also demonstrating complacency with the limited steps taken by companies continues in subsequent years. For example, in 2014, CalSTRS questions “why so much shareholder capital is going towards exploration for additional reserves, rather than being directed towards developing alternative energy sources, or being given back to shareholders” (CalSTRS, 2014a, p. 42). However, to date, little progress has been made on the issue of capital expenditures being allocated to exploration or new extraction (CA100+, 2023), and despite its criticality for limiting fossil fuel supply, this issue is not explicitly discussed again in pension fund documentation of their engagements with fossil fuel companies.

Even as pension fund engagement with fossil fuel companies widens in scope and becomes more systematic post-Paris Agreement, the content of these engagements can still be considered lacking in ambition. Though it is possible that their private discussions with companies are more ambitious, public forms of engagement (generally shareholder resolutions) consist largely of requests for disclosure (of the company’s emissions, climate strategies, etc.). Some of the pension funds explicitly state their unwillingness to support resolutions which would dictate concrete emissions reduction targets for fossil fuel companies. For example, CalPERS does not support “proposals intended to substitute for management’s operational judgements” (CalPERS, 2019, p. 15). In the last several years, as frustration with the limited evidence of change from fossil fuel companies has grown, pension funds have begun to express their discontent through voting against directors and board members, rather than through supporting or proposing more ambitious shareholder resolutions. There is little evidence to date on whether votes against directors is effective for shifting fossil fuel company strategies, although in the case of Exxon, there seems to have been little impact on Exxon’s climate strategies (Herbst-Bayliss et al., 2023). Most pension funds, with the exceptions of PFZW (since 2022) and ATP, have not supported the annual shareholder resolutions submitted by Follow This which ask for concrete emissions reduction targets. ABP shifted their strategy to divestment, without exploration of a more ambitious engagement strategy. In conceptualising the role for investors as agents of climate action, these findings thus suggest two divergent roles. On the one hand, their reluctance to act beyond the confines of the traditional shareholder role (whether that is due to norms within the fund, or restrictions based on interpretations of fiduciary duty) means their capacity for climate action is limited. At best, they may contribute to obtaining greater levels of disclosure from companies, ‘filling in the gaps’ left by disclosure regulations (Banda, 2018). At worst, their public commitment to engagement with fossil fuel companies, which gives the appearance of action, could serve as a “deadly distraction” from more effective policy intervention (Baines & Hager, 2022, p. 4; McDonnell et al., 2022). On the other hand, the willingness from two pension funds (amongst others not addressed here) to support more ambitious resolutions suggests there could be room for a redefinition of the traditional role for shareholders in the context of fossil fuels and the climate crisis, although this certainly remains a minority position.

While fossil fuel companies remain a target of shareholder engagement, there is some very recent evidence that investors are returning to prioritising demand-side engagements (Gambetta, 2023). For example, after committing to fossil fuel divestment, ABP has stated they will prioritise engagements with major fossil fuel consumers, including utilities, transport, industrial, and financial sectors (ABP, 2022b). Although this raises questions for how attention to supply-side fossil fuel restriction is prioritised, engagement with the financial sector in particular may offer new opportunities for limiting fossil fuel supply, given the fossil fuel industry’s reliance on loans for much of their expansion activities (Cojoianu et al., 2021) and the fact that a small number of asset managers and investment banks hold an outsize share of the fossil fuel sector’s assets (Dordi et al., 2022).

Amongst the strategies and policies cited by the supply-side fossil fuel literature, divestment has most relevance for investors (Gaulin & Le Billon, 2020; Pellegrini & Arsel, 2022; Piggot et al., 2020). This analysis finds clear evidence that divestment is a strategy being actively employed by the pension funds studied, although they most often adopt selective divestment policies, excluding certain industries, or the worst offenders within an industry. Divestment decisions are often discussed alongside pension commitments to reduce their portfolio emissions– indeed, divestments from the highest-emitting companies in their portfolio were the main contributor to some pension funds meeting their progressive emissions reduction targets (e.g. PFZW, 2020). This suggests that as pension funds come under pressure to meet their own net zero goals, divestments could increase. This is in line with the findings of other studies which indicate that reducing portfolio exposure to high-carbon sectors and companies is a strategy being pursued by investors, especially those which track their carbon emissions (Boermans & Galema, 2019; Bolton & Kacperczyk, 2021; Egli et al., 2022). However, the impact of such divestments on the fossil fuel sector and on actual restrictions to fossil fuel supply remain unknown. The risk of merely transferring the problem of fossil fuel assets to another, potentially less transparent, actor should not be ignored and merits ongoing research (Christophers, 2021).

Finally, this analysis suggests that conceptualising the role of pension funds in climate action may require broadening consideration of their scope of influence beyond that of shareholders alone to include political influence. Pension funds have a long history of lobbying for the adoption of climate policy, or specific regulations, although few specifics of the contents or details of their engagement with policymakers are known. The main insight available from pension fund reporting is on the spaces in which and types of policies for which they are lobbying. They indicate their support for four main types of policy: general international climate agreements, increased disclosure requirements for companies, carbon pricing, and phasing out fossil fuel subsidies. Golka and van der Zwan (2022) argue that in an increasingly financialised economy, there is a growing niche for financial experts to exercise influence in policy spaces, which in turn shapes the types of policies adopted. Although the analysis in this paper is not sufficient to determine the level of influence pension fund lobbying has on policy choices, the types of policy that pension funds vocally support are aligned with the recommendations of many economists and international organisations, and do not demonstrate significant ambition on restricting fossil fuels. Clear policy signals and greater company disclosure are amongst preferred strategies to help investors minimise uncertainty and ambiguity, although literature indicates that improved disclosure alone will be insufficient for addressing climate change (Ameli et al., 2020; Chenet et al., 2021; Christophers, 2017). Advocating for the phaseout of fossil fuel subsidies is aligned with the policies advocated for by proponents of supply-side restrictions, while still conforming to the logic of markets and removing market distortions. Further research into pension fund influence in the policy arena could examine their alignment with other supply-side policy recommendations.

## Conclusion

Tracing the evolution of the climate strategies of the largest US and EU pensions reveals that these pensions have explicitly been considering climate factors in their investment strategies for over 20 years. This paper examines how pension fund strategies have evolved throughout three general ‘eras’ of pension action in relation to the Kyoto Protocol, the Copenhagen Accord, and the Paris Agreement, and analyses the implications of the development of pension fund climate strategies for supply-side fossil fuel restriction. While their climate action has grown rapidly over the last decades, there remain many limitations to the effectiveness of pension fund strategies for climate mitigation. Although pension funds often employ language which aligns with goals of fossil fuel phase-out, their response to fossil fuel company action also demonstrates complacency and acceptance of the small, often disingenuous, steps taken by fossil fuel companies (Megura & Gunderson, 2022). Significantly contributing to phasing out fossil fuels will likely require a radical rethinking and redefinition of the role for pension funds in addressing the climate crisis. As ATP points out, at present, “there is no authoritative way of determining whether investors ‘comply with’ the Paris Agreement” (ATP, 2019, p. 5). Assessing the most effective contributions investors might make, and developing policy to enable such contributions remains a pressing challenge.

## Appendix

### Document types

**Table:**

	Date range of collected reports	Annual Reports	Sustainability Reports	Stewardship/Corporate Governance Reports	Proxy Voting Guidelines	Investment policies	Other relevant publications	Total
CalPERS	1998–2022	22	4	13	7	0	16	62
CalSTRS	1994–2022	27	19	16	0	12	11	85
NYSCRF	2000–2022	23	7	7	5	5	17	64
ABP^a^	1996–2022	27	6	6	0	0	6	45
PFZW	2005–2022	18	1	11	1	0	7	38
ATP	2014–2022	8	1	17	0	0	6	32
Total		125	38	70	13	17	63	326

^a^For PFZW and ABP, corporate governance reports are produced by the administrative organisations or asset managers (PGGM and APG respectively) carrying out the pension fund mandates. These reports have also been included

### Atlas.ti search

*The following search terms were used, with the option to identify inflected forms enabled:*

“Climate OR global warming OR sustainable OR sustainability OR environment OR environmental OR environmentally OR esg OR green OR coal OR oil OR gas OR LNG OR petroleum OR energy OR fossil fuel OR fossil OR emission OR GHG OR net zero OR net-zero OR carbon OR UNFCCC OR Kyoto OR Copenhagen OR Paris”

*Dutch search terms:*

“Klimaat OR klimaatverandering OR opwarming OR duurzaam OR duurzame OR duurzaamheid OR milieu OR ESG OR verantwoord OR verantwoordelijk OR verantwoordelijke OR groen OR groene OR bruinkool OR steenkool OR olie OR aardolie OR gas OR aardgas OR vloeibaar aardgas OR benzine OR fossiel OR fossiele OR uitstoot OR uitstoten OR broeikasgas OR broeikasgassen OR energie OR koolstof OR GHG OR netto nul OR klimaatneutraliteit OR CO2 OR UNFCCC OR Kyoto OR Kopenhagen OR Parijs,”

## Abbreviations

ABP: Algemeen Burgerlijk Pensioenfonds
ATP: Arbejdmarkedets Tillægspension
CA100+: Climate Action 100+
CalPERS: California Public Employees Retirement System
CalSTRS: California State Teachers Retirement System
ESG: Environment, Social, and Governance
NYSCRF: New York State Common Retirement Fund
NZAOA: Net Zero Asset Owner Alliance
PFZW: Pensioenfonds Zorg & Welzijn
PRI: Principles for Responsible Investment
SEC: Securities and Exchange Commission
TCFD: Task Force on Climate-Related Financial Disclosures
UNFCCC: UN Framework Convention on Climate Change
INCR: Investor Network on Climate Risk

## Footnotes

1 https://archive.org/

2 https://publicplansdata.org/
